# Pump‐Induced Hemolysis of Speed Modulated Axial‐Flow Left Ventricular Assist Devices

**DOI:** 10.1111/aor.14966

**Published:** 2025-02-17

**Authors:** Patrick Borchers, Ailín Österlein Kück, Steffen Leonhardt, Marian Walter

**Affiliations:** ^1^ Chair for Medical Information Technology, Helmholtz Institute for Biomedical Engineering RWTH Aachen University Aachen Germany

**Keywords:** axial‐flow pump, HeartMate2, hemolysis, left ventricular assist device, mock circulatory loop, speed modulation, Sputnik1

## Abstract

**Background:**

Reduced arterial pulsatility during continuous‐flow left ventricular assist device (LVAD) support is associated with certain adverse events. An approach to increase arterial pulsatility is pump speed modulation. Therefore, this in vitro study compares the pump‐induced hemolysis of constant speed and modulated speed modes for two different axial‐flow LVADs. Furthermore, the hemolytic performance of both LVADs is compared.

**Methods:**

Two Sputnik1 and two HeartMate2 (HM2) axial‐flow LVADs were operated simultaneously for 6 h in automated hemolysis test benches (*n* = 7 for each mode). Rectangular speed patterns with modulation rates of 70 and 140 bpm were investigated. Speed modulation amplitudes provided head pressures between 80 and 120 mmHg. To quantify hemolysis, plasma‐free hemoglobin was determined every hour and the modified index of hemolysis (MIH) was calculated.

**Results:**

Speed modulation increased all MIH values of the Sputnik1 LVADs, but decreased most MIH values of the HM2 LVADs compared to the constant speed mode. However, significant differences were only observed for one Sputnik1. Furthermore, the Sputnik1 pumps induced lower MIH levels compared to the HM2 pumps using constant speed mode.

**Conclusions:**

It seems that the HM2 can be operated in speed modulation mode without an increased risk of hemolysis. However, for the Sputnik1, the potential benefits of speed modulation must be balanced against the risk of increased hemolysis. The underlying causes need to be investigated in future studies using computational fluid dynamics. Furthermore, using the clinically established constant speed mode, it appears that the Sputnik1 will cause fewer hemolytic issues than the HM2.

## Graphical abstract

Two Sputnik1 and two HeartMate2 axial‐flow LVADs were operated in hemolysis test benches under both constant speed and speed modulation modes (*n* = 7). One of the Sputnik1 pumps exhibited a statistically significant increase in hemolysis, while none of the HeartMate2 pumps showed statistically significant differences.

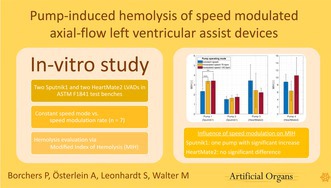

## 1 Introduction

The use of implantable left ventricular assist devices (LVADs) has been demonstrated to enhance survival and quality of life in patients with end‐stage heart failure [1]. These devices are used to bridge patients to heart transplantation or as a permanent solution for those who are not eligible for transplantation [2]. Today, continuous‐flow LVADs have mostly replaced the first‐generation pulsatile‐flow LVADs due to higher survival rates, smaller size, improved reliability, and durability [3]. According to the Interagency Registry for Mechanically Assisted Circulatory Support (Intermacs) [2], about 27 000 patients received an isolated continuous‐flow LVAD from January 2012 until December 2021, accounting for 93.7% of all implanted mechanical circulatory support devices. Patients supported by continuous‐flow LVADs already demonstrate 2‐year survival rates over 70% [4], which has led to an increasing use of these devices for destination therapy. While only 46.6% of continuous‐flow LVADs were used for destination therapy in 2014, this portion increased to 80.1% in 2019 [5].

For long‐term support, hemocompatibility of LVADs is especially important [6]. Continuous‐flow LVADs employ a rotating impeller to pump blood, thereby subjecting blood cells to high shear stress [7]. This causes hemolysis, which is the leakage of red blood cell content into the plasma due to the formation of pores or the lysis of red blood cells [8]. Severe hemolysis can result in hemolytic events, which are associated with significantly decreased survival rates [9, 10]. This is likely caused by the linkage between hemolysis and thrombus formation [9, 10].

Furthermore, certain adverse complications like gastrointestinal bleeding, arteriovenous malformations, pump thrombosis, and aortic insufficiency, reduced ventricular unloading, and lower rates of myocardial recovery [3, 11, 12, 13] are thought to be related to the diminished arterial pulsatility during continuous‐flow LVAD support [3, 11]. One strategy to augment the circulatory pulsatility is to use a modulated speed (MS) mode instead of the traditional constant speed (CS) mode. Here, the LVAD generates periodic speed steps, synchronously or asynchronously to the heart cycle. However, whether pump speed modulation is desirable or not is still under active debate [12, 13]. According to Schima et al. [12], only a limited increase in pulsatility can be achieved with current devices and potential risks must be excluded before introducing speed modulation into clinical practice. One potential risk is an increase in LVAD‐induced hemolysis due to the rapid change in impeller speed [12]. Nevertheless, it is also possible that speed modulation may reduce hemolysis by improving pump washout, thus preventing stasis zones [13].

A few in vitro hemolysis studies already compared CS and MS modes of continuous‐flow LVADs [14, 15, 16, 17, 18, 19]. These studies are summarized in Table 1, listing the investigated LVAD, the used operating conditions, the number of trials per mode, and the induced hemolysis of CS and MS modes. Most studies observed a statistically insignificant increase in pump‐induced hemolysis when using MS mode compared to CS mode [15, 16, 18, 19]. Yet, Kishimoto et al. [14] found no difference, and Rungsirikunnan et al. [17] even observed a decrease using a single trial per operating mode. We note that all the listed studies were conducted using centrifugal‐flow LVADs. To the best of our knowledge, no in vitro hemolysis studies have been published yet comparing CS and MS modes of axial‐flow LVADs. Due to the distinct design characteristics of axial‐flow LVADs, findings from centrifugal‐flow LVADs cannot be directly extrapolated to these devices. Therefore, this study investigated whether the rapid acceleration and deceleration during speed modulation influence pump‐induced hemolysis of axial‐flow LVADs. The landmark HeartMate2 (HM2) and the recently introduced Sputnik1 LVAD have been selected for this purpose. Although the centrifugal‐flow LVAD HeartMate3 has largely replaced the landmark HM2 LVAD in clinical practice [2], access to Western medical devices remains limited in certain regions. In particular, there is growing interest in the Asia‐Pacific region in lower‐cost alternatives to make LVAD therapy accessible to a broader patient population [20]. One such alternative is the Sputnik1 LVAD, which is currently in clinical use in Russia [21]. The HM2 was included in this study because it remains a widely studied LVAD, making it a valuable benchmark for comparing newer axial‐flow devices, such as the Sputnik1 pump, and for evaluating the effects of the specific pump design on speed modulation. A secondary objective of this study was to compare the degree of hemolysis caused by both LVADs using the same experimental setup, which may provide valuable insights for improving specific design features of axial‐flow LVADs.

**TABLE 1:** Studies on speed modulation of continuous‐flow LVADs.

Author/Article	LVAD	Operating mode	Rotational speed [rpm]	Pressure head [mmHg]	Pump flow [L/min]	Modulation rate [bpm]	Number of trials	NIH [g/100 L], MIH [.] or HRI [g/h] (mean ± SD)
Tayama et al. [18]	Gyro C1E3 (centrifugal)	CS	1605	100	5	40	4	NIH: 0.0010 ± 0.0003
		MS	1763–1234	69–123	4.2–5.2^a^			NIH: 0.0014 ± 0.0006
Kono et al. [15]	Unspecified (centrifugal)	CS	1900	100	5	75	4	NIH: 0.0025 ± 0.0006
		MS	1700–2100	70–130^a^	3.5—6.2			NIH: 0.0032 ± 0.0009
Kishimoto et al. [14]	EVAHEART (centrifugal)	CS	2352	100	5	60	4	NIH: 0.0023 ± 0.0025
		MS	2169–2679	85–135^a^	4.5—5.9			NIH: 0.0023 ± 0.0019
Naito et al. [16]	EVAHEART (centrifugal)	CS	2357	100	5	60	4	NIH: 0.0031 ± 0.0007
		MS	2045–2545	—	—			NIH: 0.0035 ± 0.0018
Rungsirikunnan et al. [17]	MUPD VAD (centrifugal)	CS	1500	60	3.2	60	1	MIH: 23.42
		MS	1250–1750	47–73	2.9–3.5			MIH: 22.04
		MS	1000–2000	41–79	2.7–3.7			MIH: 22.05
Horobin et al. [19]	HVAD (centrifugal)	CS	3282	90	5	60	7	HRI: 0.157 ± 0.037^a^
		MS	2843–3703	80–121	—			HRI: 0.201 ± 0.037^a^

*Note:* For each study, the utilized LVAD, the investigated operating conditions, and the number of trials are listed. Depending on the study, the normalized index of hemolysis (NIH), the modified index of hemolysis (MIH), or the hemolysis rate index (HRI) were determined as hemolysis indices. The hemolysis indices are reported for the constant speed (CS) mode and the modulated speed (MS) mode, respectively.

^a^ Determined from graphs.

## 2 Methods

### 2.1 Investigated LVADs

Two Sputnik1 (National Research University of Electronic Technology and others, Moscow, Russia) and two HM2 axial‐flow LVADs (Abbott Laboratories, Chicago, USA) were used in this in vitro hemolysis study. In the following text, the two Sputnik1 LVADs are designated as Pump 1 and Pump 2, while the HM2 LVADs are designated as Pump 3 and Pump 4. Detailed information on the Sputnik1 pump can be found in [22] and on the HM2 pump in [23]. In short, both pumps consist of a fixed stator and diffusor with three blades each. A magnetic impeller rotor, with four blades for Sputnik1 and three blades for HM2, is located between needle bearings and driven by coils in the pump housing. Both LVADs have a pump length of 81 mm. The Sputnik1 LVAD has a weight of 200 g, while the HM2 pump has a weight of 290 g. The typical clinical operating speed of the Sputnik1 is 6000–8000 rpm [24] and of the HM2 is 8600–9800 rpm [25]. Until the beginning of 2020, the Sputnik1 has been implanted in 49 patients [21], whereas the HM2 has been implanted more than 27 000 times by the end of 2023 [26].

### 2.2 Automated Hemolysis Test Benches

**FIGURE 1:**
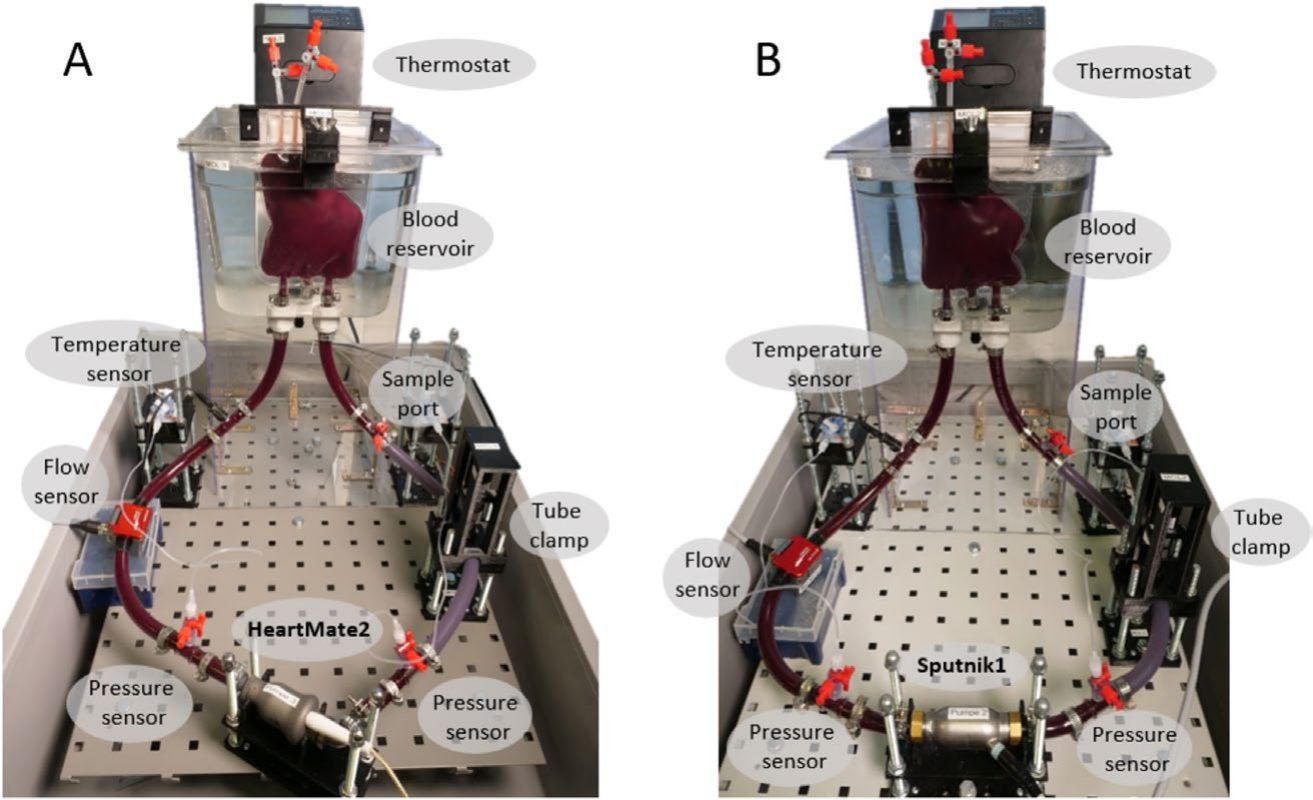
Automated hemolysis test bench equipped with a HeartMate2 (A) and a Sputnik1 LVAD (B). [Color figure can be viewed at wileyonlinelibrary.com]

Each LVAD was assigned to a hemolysis test bench according to the ASTM standard [27]. In this study, the utilized test benches (c. f. Figure 1) were able to automatically set and maintain predefined LVAD operating conditions, thereby reducing the workload and improving the reproducibility of the experiments. Moreover, axial‐flow LVADs can be operated with arbitrary speed modulation patterns. All four test benches were connected to a dSPACE MicroLabBox system (dSPACE GmbH, Paderborn, Germany) for synchronous recording of the operating conditions and for controlling the actuators. The dSPACE box created the reference speed signal for a Maxon 50/4 motor driver module (Maxon Motor AG, Sachseln, Switzerland), which performed closed‐loop speed control of the LVADs. The required hydraulic resistance, which mimicked the total peripheral resistance, was generated by a self‐developed controllable tube clamp. The clamp consists of an Actuonix P16 (256:1) linear actuator (Actuonix Motion Devices Inc., Saanichton, Canada) driven by a Pololu G2 high‐power motor driver (Pololu Corporation, Las Vegas, USA). Inlet and outlet pump pressures were measured using Xtrans DPT‐9300 pressure sensors (Codan pvb Medical GmbH, Lensahn, Germany). The pump flow was measured using a Transonic HT‐110 (Transonic Systems Inc., New York, USA) or a Sonoflow CO.55/120 (Sonotec GmbH, Halle, Germany) clamp‐on sensor. A 400 mL Capiox Flexible Venous Reservoir (Terumo Corporation, Tokyo, Japan) was immersed in a water bath, which was heated to 37°C using an Allpax SV2 thermostat (Allpax GmbH & Co. KG, Papenburg, Germany). The blood temperature was monitored by an NTC sensor, which accompanies the blood reservoir. Raumedic ECC‐noDOP 1/2 × 3/32 tubes and ECC‐Silicon 1/2 × 3/32 tubes (Raumedic AG, Münchberg, Germany) as well as polycarbonate connectors (Fleima‐Plastic GmbH, Wald‐Michelbach, Germany) were utilized. Three‐way valves (BBraun SE, Melsungen, Germany) were used as sample ports. Each test bench had a blood volume of 450 mL.

The four test benches automatically started one after another with a 3‐min delay to allow for sample processing in between. During the initial 9 min, the blood was mixed and preheated using a pump flow of 5 L/min with an opened clamp. Afterwards, the desired mean operating conditions of head pressure (outlet minus inlet pressure) and pump flow were automatically set within 1 min. Subsequently, the actual 6‐h experiment commenced, where the desired speed modulation patterns were superimposed onto the required mean pump speed. Additional information on modeling and control of the test benches can be found in [28].

### 2.3 Study Design

Constant speed mode (CS), modulated speed mode with a modulation rate of 70 bpm (MS70), and modulated speed mode with a modulation rate of 140 bpm (MS140) were investigated in this study (c. f. Table 2). 70 bpm corresponds to the heart rate of LVAD patients at rest, whereas 140 bpm corresponds to the heart rate of LVAD patients at peak exercise [29]. For both MS modes, a rectangular speed pattern was utilized as it corresponds to the highest possible (worst‐case) acceleration rate. However, to avoid pressure oscillations during speed changes and minimize the risk of pump stoppage, the rectangular speed pattern was filtered with a 20 Hz low‐pass filter. To achieve head pressure levels between 80 and 120 mmHg, the modulation amplitude for Sputnik1 was set to 1000 rpm and for HM2 was set to 1150 rpm, respectively. Consistent with the majority of studies presented in Table 1, the mean flow was controlled to 5 L/min and the mean head pressure to 100 mmHg for all modes.

**TABLE 2:** Investigated LVAD operating modes.

Operating mode	Mean pump flow [L/min]	Mean head pressure [mmHg]	Systolic/Diastolic head pressure [mmHg]	Modulation rate [bpm]	Modulation amplitude [rpm]	Modulation shape
Constant speed (CS)	5	100	—	—	—	—
Modulated speed; 70 bpm (MS70)	5	100	80/120	70	HeartMate2: 1150 Sputnik1: 1000	Rectangular (20 Hz filtered)
Modulated speed; 140 bpm (MS140)	5	100	80/120	140	HeartMate2: 1150 Sputnik1: 1000	Rectangular (20 Hz filtered)

Seven trials were conducted for each LVAD under each operating mode. During each trial, fresh porcine blood from the same pool, anticoagulated with 6000 international units of heparin per liter (B. Braun, Melsungen, Germany), was circulated for 6 h in all four test benches. The hematocrit was adjusted to 35% by diluting the blood with phosphate‐buffered saline solution (Merck KGaA, Darmstadt, Germany), and the glucose level was adjusted to 6 mmol/L by adding glucose (VWR International, Pennsylvania, USA). After each trial, the pumps and the test benches were cleaned with a solution of pepsin and citric acid (AppliChem GmbH, Darmstadt, Germany).

### 2.4 Blood Sampling and Hemolysis Determination

Blood samples were collected hourly from each test bench. After discarding 1 mL of stagnant blood, 2 mL of blood were withdrawn for further analysis. To assess hemolysis, plasma‐free hemoglobin (pfHb) was determined with the cyan hemoglobin method using a dedicated reagent (Bioanalytic GmbH, Umkirch, Germany) according to the manufacturer's instructions. The blood was first centrifuged for 15 min at 2000 rcf, and the extracted plasma was then again centrifuged for 15 min at 13000 rcf. The 4:1 mixture of reagent and plasma was analyzed with a PV4 UV/VIS spectrophotometer (Avantor, Pennsylvania, USA) using the two‐wavelength method according to Taperon (540/680 nm). Furthermore, hematocrit, total hemoglobin concentration, red blood cell count (RBC), white blood cell count (WBC) and platelet count (PLT) were measured using the MEK‐6550 K cell counter (Nihon Khoden Inc., Rosbach, Germany). Moreover, oxygen saturation, pH, lactate, and potassium concentration were measured every 2 h using an ABL800 blood gas analyzer (Radiometer Inc., Fichtenhain, Germany). Blood glucose was measured using the ABL800 or an Accu‐Chek Guide device (Roche Holding AG, Basel, Switzerland).

The modified index of hemolysis (MIH) of each 1‐h interval was calculated according to the ASTM standard [27]:

(1) $$\mathrm{MIH}\;\left[-\right]=\frac{\text{ΔpfHb}\cdot V\cdot \frac{\left(100-\mathrm{HCT}\right)\;}{100}}{Q\cdot \Delta T\cdot \mathrm{Hb}\;}\cdot 10^{6}$$

In this formula, Δ*T* is the duration between two consecutive blood samples [min] and ΔpfHb is the change in pfHb [mg/dL] during each interval. HCT is the hematocrit [%], Hb is the total hemoglobin concentration [mg/dL], *V* is the blood volume of the test bench [L], and *Q* is the mean blood flow [L/min] during each interval. The overall MIH value for each trial was then determined by averaging across the six individual 1‐h intervals. The normalized index of hemolysis (NIH) according to [27] was also determined. However, since NIH does not account for hemoglobin concentration and does not show significant differences in trends compared to the MIH, it is only included in the Data S1.

### 2.5 Statistical Analysis

Statistical analysis was performed with Matlab 2023a. All data are presented as mean ± SD. The statistical significance was evaluated using two‐sided two‐sample Welch's *t*‐tests. Only *p*‐values less than 0.05 were considered statistically significant (*p < 0.05; **p < 0.01; ***p < 0.001).

## 3 Results

### 3.1 Operating Conditions

The measured operating conditions for all operating modes and all LVADs are presented in Table 3. The mean flow and the mean head pressure were closely regulated to 5 L/min and 100 mmHg for all cases. Furthermore, the mean speed and mean blood temperature were similar between the operating modes. Notably, no significant difference in mean power consumption was observed between CS and MS modes, indicating that pulsatile operation does not impose a considerable penalty on power efficiency.

**TABLE 3:** Measured LVAD operating conditions for all operating modes and all pumps.

Operating mode	Mean flow [L/min]	Mean pressure head [mmHg]	Mean speed [rpm]	Mean power [W]	Mean blood temperature [°C]
Pump 1 (Sputnik1)					
CS	5.00 ± 0.02	99.9 ± 0.5	8814 ± 33	18.3 ± 0.3	36.9 ± 0.2
MS70	5.00 ± 0.02	99.9 ± 0.5	8815 ± 27	18.4 ± 0.3	36.9 ± 0.3
MS140	5.00 ± 0.03	100.0 ± 0.5	8809 ± 44	17.7 ± 1.7	37.0 ± 0.2
Pump 2 (Sputnik1)					
CS	5.00 ± 0.02	99.9 ± 0.6	8955 ± 42	18.1 ± 2.0	36.9 ± 0.2
MS70	5.00 ± 0.02	100.0 ± 0.8	8858 ± 46	18.2 ± 0.6	36.9 ± 0.2
MS140	5.00 ± 0.02	100.0 ± 0.6	8926 ± 32	18.4 ± 0.8	36.9 ± 0.2
Pump 3 (HM2)					
CS	5.00 ± 0.05	99.9 ± 1.2	10 985 ± 135	15.7 ± 1.5	36.0 ± 0.3
MS70	5.00 ± 0.02	99.9 ± 0.5	10 929 ± 109	16.1 ± 1.8	36.1 ± 0.3
MS140	5.00 ± 0.03	100.0 ± 0.7	10 992 ± 101	15.3 ± 2.1	36.2 ± 0.2
Pump 4 (HM2)					
CS	5.00 ± 0.02	100.0 ± 0.7	10 908 ± 87	15.2 ± 2.0	36.3 ± 0.2
MS70	5.00 ± 0.02	100.0 ± 0.5	10 880 ± 65	15.9 ± 1.9	36.2 ± 0.3
MS140	5.00 ± 0.02	100.0 ± 0.6	10 924 ± 76	15.5 ± 1.4	36.1 ± 0.2

Comparing Sputnik1 and HM2, the Sputnik1 pumps required a mean speed of about 8850 rpm to produce the intended operating conditions, whereas the HM2 pumps required about 10 950 rpm. Furthermore, the Sputnik1 pumps required about 2.6 W more power compared to the HM2 pumps and the blood temperature of the Sputnik1 pumps was about 0.8°C higher.

**FIGURE 2:**
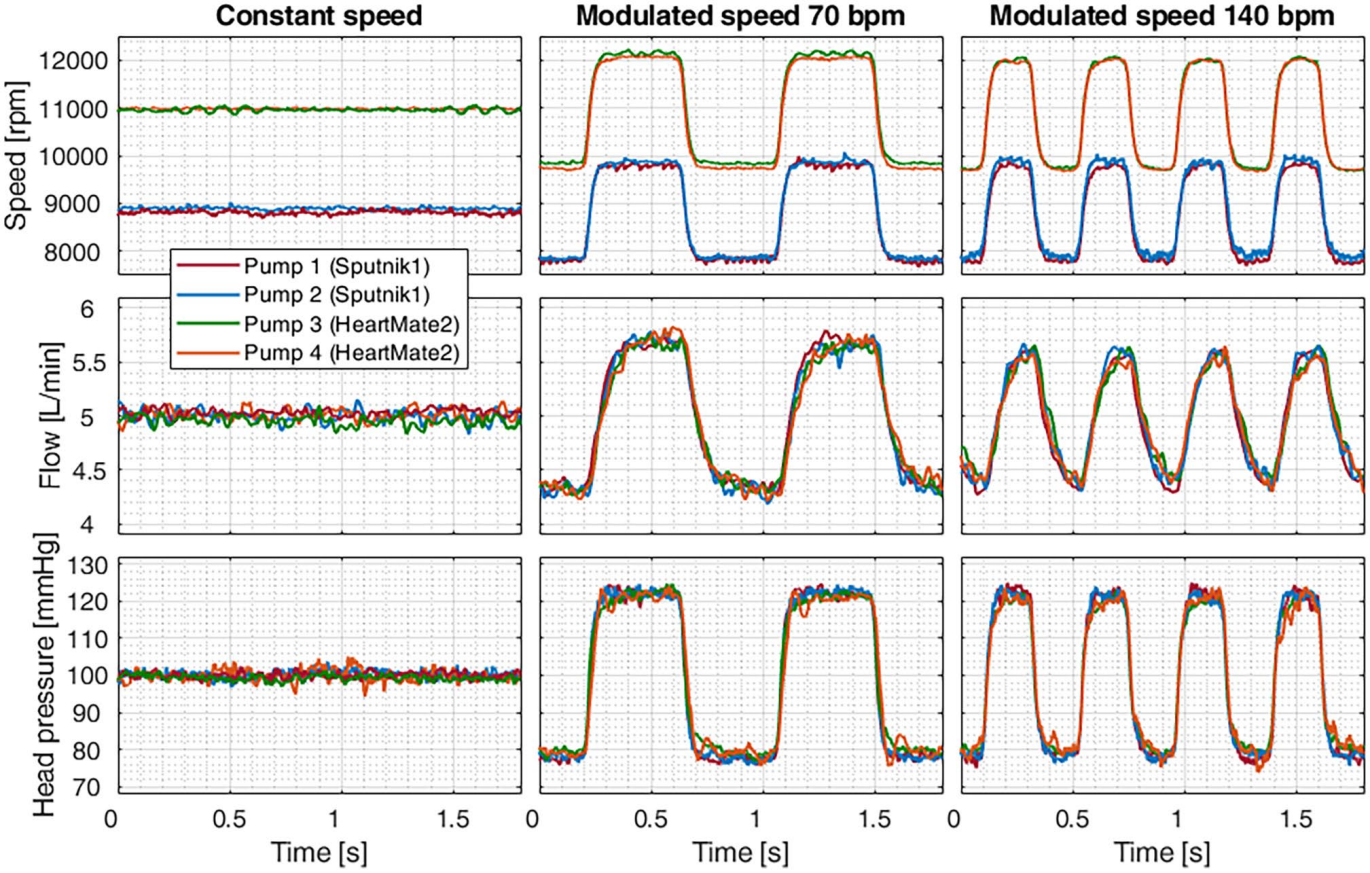
Recorded speed, flow, and head pressure signals of all pumps operated at all operating modes. [Color figure can be viewed at wileyonlinelibrary.com]

The recorded speed, flow, and head pressure signals for the CS and the two MS modes are exemplarily shown in Figure 2. We note that the head pressure and flow signals are comparable for all pumps. As intended, the diastolic head pressure is about 80 mmHg, and the systolic head pressure is about 120 mmHg for the MS modes. This results in flow variations between 4.3 and 5.7 L/min.

### 3.2 Hemolysis

**FIGURE 3:**
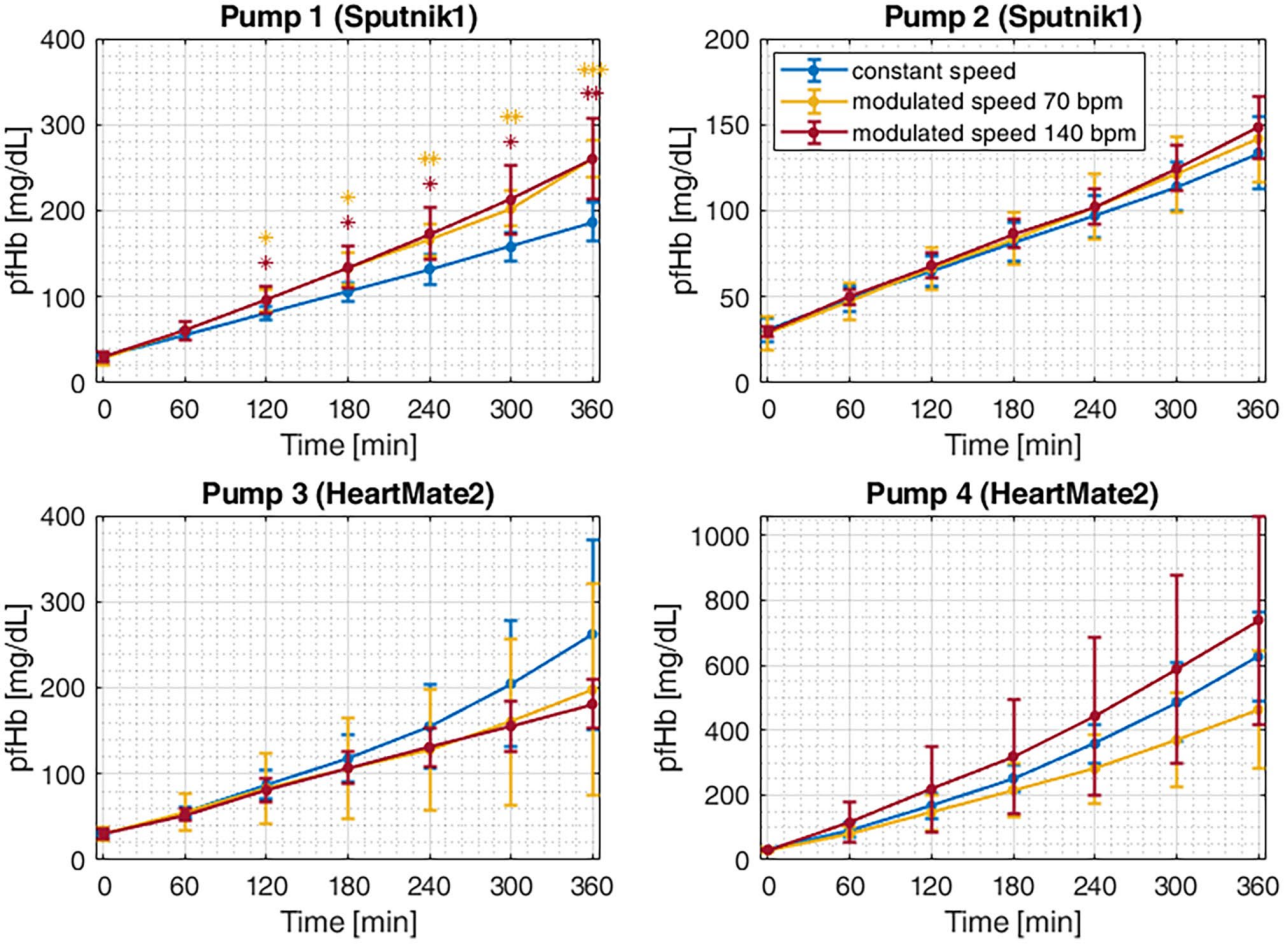
Plasma‐free hemoglobin (pfHb) over time for all operating modes and all pumps. Significance was tested for the CS mode compared to both MS modes. *p < 0.05; **p < 0.01; ***p < 0.001. [Color figure can be viewed at wileyonlinelibrary.com]

The temporal evolution of pfHb for all LVADs and all operating modes is shown in Figure 3. Significant differences between CS and the MS modes were only revealed for pump 1. The MS modes showed significantly increased pfHb values after at least 120 min, with increasing significance levels as the test time progressed.

**FIGURE 4:**
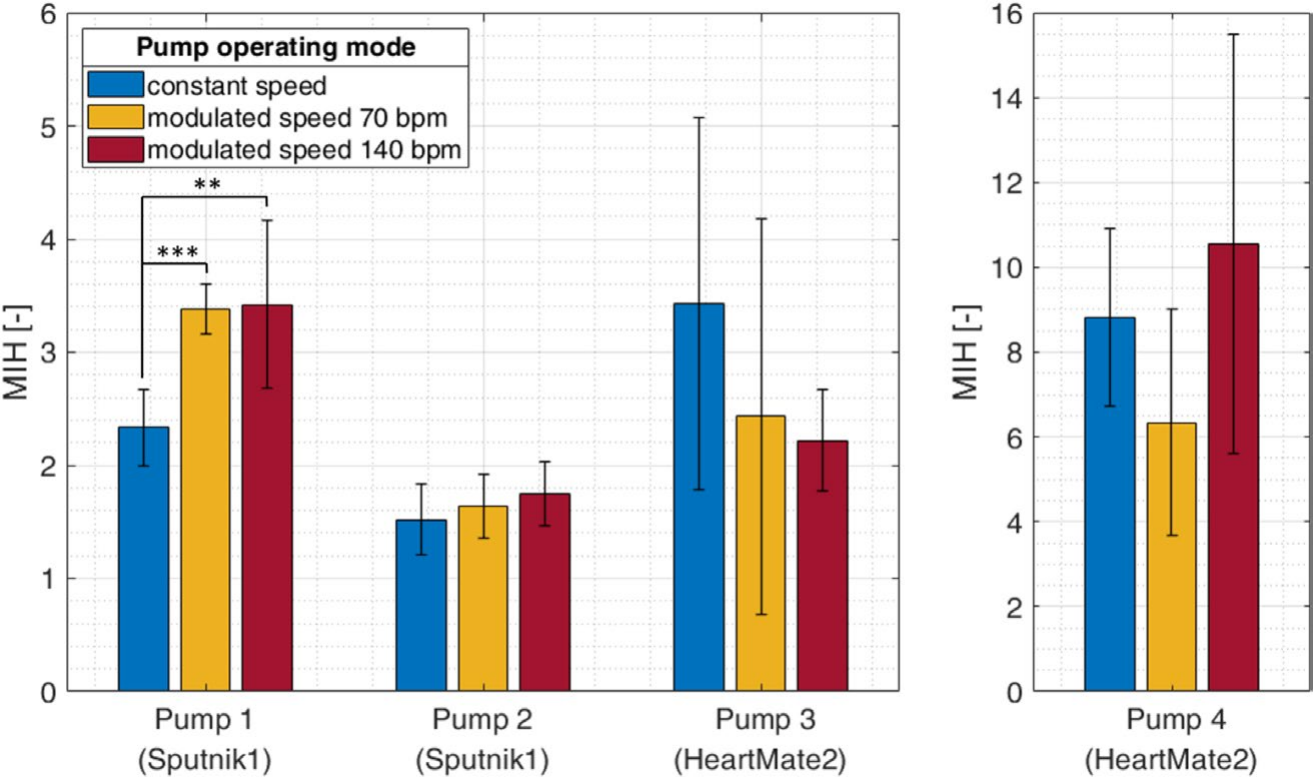
The modified index of hemolysis (MIH) for all operating modes and all pumps. Significance was tested for the CS mode compared to both MS modes. *p < 0.05; **p < 0.01; ***p < 0.001. [Color figure can be viewed at wileyonlinelibrary.com]

For all groups, the MIHs are presented in Figure 4. Significant differences were only observed for Pump 1. The MIH for Pump 1 significantly increased by 44.7% (*p* < 0.001) for MS70 and by 46.6% (*p* = 0.007) for MS140 compared to the CS mode. For Pump 2, the MIHs were also higher for the MS modes. However, this increase was only 7.9% (*p* = 0.47) for MS70 and 15.4% (*p* = 0.17) for MS140. In contrast, the speed modulation of Pump 3 resulted in a decrease of MIH by 29.1% (*p* = 0.29) for MS70 and by 35.3% (*p* = 0.10) for MS140. For Pump 4, there was a decrease of 28.0% (*p* = 0.08) for MS70 and an increase of 19.6% (*p* = 0.42) for MS140.

Additionally, the hemolytic performance of the Sputnik1 and the HM2 LVAD can be compared. First of all, Pump 4 showed significantly higher MIHs compared to both Sputnik1 LVADs for all operating modes (*p* < 0.03). For Pump 3, the comparison to the Sputnik1 LVADs depends on the operating mode. In CS mode, Pump 3 showed no significant difference to Pump 1 (*p* = 0.13), but a significantly higher MIH compared to Pump 2 (*p* = 0.02). For MS70, no significant difference was observed between Pump 3 and the Sputnik1 LVADs. For MS140, Pump 3 caused a significantly lower MIH compared to Pump 1 (*p* = 0.005), but a significantly higher MIH compared to Pump 2 (*p* = 0.04).

### 3.3 Further Blood Parameters

Further blood parameters over time are presented in the Data S1. No significant differences between the operating modes were observed for HCT, WBC, pH, lactate, and potassium concentration. Only at a few time instances, statistically significant differences with *p* > 0.01 were observed for Hb, RBC, PLT, glucose, and oxygen saturation.

## 4 Discussion

The primary objective of this in vitro study was to analyze whether speed modulation of axial‐flow LVADs affects pump‐induced hemolysis. To minimize variability due to wear or pre‐damage, the effect of speed modulation was analyzed separately for each LVAD. Speed modulation tended to increase the hemolysis induced by the Sputnik1 pumps, though only one of the two tested pumps showed significant differences. This trend is consistent with the majority of studies on centrifugal‐flow LVADs presented in Table 1. Additionally, it aligns with computational fluid dynamics (CFD) simulations of the Sputnik1 LVAD by Romanova et al. [30], which estimated a 30.6% increase in the hemolysis index using speed modulation. Conversely, MS modes tended to decrease the hemolysis induced by the HM2 pumps, though neither of the two HM2 devices exhibited statistically significant differences. Consequently, this study does not raise concerns about using speed modulation with the HM2 pumps in terms of hemolysis. For the Sputnik1, however, the potential benefits of speed modulation must be balanced against the risk of increased hemolysis. The opposing (but non‐significant) trends observed between Sputnik1 and HM2 raise the possibility that the effect of speed modulation could be influenced, at least in part, by differences in the specific design of these LVADs.

The significant increase in hemolysis for Pump 1 (Sputnik1) might be caused by additional shear stress due to the rapid acceleration and deceleration of the impeller speed. However, since there were twice as many speed changes for 140 bpm compared to 70 bpm, but only a slight difference in hemolysis was observed for Pump 1, this difference might also be caused by switching between the systolic and diastolic operating points. Thereby, one operating point causes more damage than the other causes less. This interpretation is also supported by the CFD simulations of Romanova et al. [30]. A similar explanation might account for the non‐significant decrease in hemolysis observed for Pump 3 (HM2). To better identify the underlying causes, such as altered flow profiles, pump washout, or shear stresses, future studies could conduct CFD simulations for the HM2 or use advanced measurement techniques like particle flow velocimetry.

As a secondary objective, pump‐induced hemolysis caused by the Sputnik1 and the HM2 can be directly compared using the same experimental setup and procedures. For the established CS mode, the Sputnik1 LVADs caused less hemolysis. However, when speed modulation was used, a superior pump type could no longer be identified. The limited number of specimens per pump type (*n* = 2) and the high variability in MIH constrain the strength of this direct comparison.

Limitations: To remain consistent with the studies on centrifugal‐flow LVADs referenced in Table 1, a simplified hemolysis test bench following the ASTM F1841 standard was used. However, due to the minimal compliance compared to the aorta and a lack of the heart's residual contractility, the pressure and flow waveforms are not physiologically translatable. Consequently, achieving a pulse pressure of 40 mmHg through speed modulation in LVAD patients would require slower modulation rates (about 20 bpm) than used in this study [31]. Furthermore, the history of the utilized LVADs prior to this study is unknown. Due to the high variances of the HM2 pumps, the conclusions drawn from these pumps must be treated with caution. Furthermore, the fact that Pump 4 caused more than twice as much hemolysis compared to Pump 3 could be attributed to prior damage or wear to the mechanical bearings. Nevertheless, as LVADs are typically in use for several months or even years, some degree of wear or damage also occurs in clinical practice. Furthermore, porcine blood was used instead of human blood. However, porcine blood is accepted by the ASTM F1841 standard and, according to Ding et al. [32], it behaves similarly to shear stress under device‐relevant conditions. In addition, the LVADs were operated at a slightly higher speed than in the clinical setting. However, 5 L/min and 100 mmHg were chosen as operating points to maintain consistency with previous studies. Moreover, the small number of trials (*n* = 7) is another limitation, although it is higher than in the majority of studies presented in Table 1. Future studies should expand their focus beyond hemolysis to include additional parameters, such as von Willebrand factor degradation and platelet damage as an indicator of gastrointestinal bleeding, as well as platelet activation as a marker of thrombus formation.

## Author Contributions

Concept/design: P.B., M.W.; Data collection and statistics: P.B., A.Ö.K.; Data analysis/interpretation: P.B.; Drafting the article: P.B.; Critical revision and approval of the article: P.B., A.Ö.K., S.L., M.W.; Funding secured by: S.L., M.W.

## Conflicts of Interest

The authors declare no conflicts of interest.

## Supporting information


Data S1.
