# Supplementary material for: Pump‐Induced Hemolysis of Speed Modulated Axial‐Flow Left Ventricular Assist Devices
Source: Artif Organs. 2025 Feb 17;49(6):988–96. doi: 10.1111/aor.14966 (PMC12120808; doi:10.1111/aor.14966)
Supplement: Supplementary file 1 — Data S1. [file AOR-49-988-s001.docx]

Supplementary Material

1. Normalized index of hemolysis (NIH)

| $\mathbf{NIH}\left[ \frac{\boldsymbol{g}}{\boldsymbol{100 L}} \right]\mathbf{=}\frac{\boldsymbol{\Delta pfHb\cdot V\cdot}\frac{\left( \mathbf{100-HCT} \right)\text{ }}{\mathbf{100}}}{\boldsymbol{Q\cdot\Delta T}}$ | (1) |
| --- | --- |


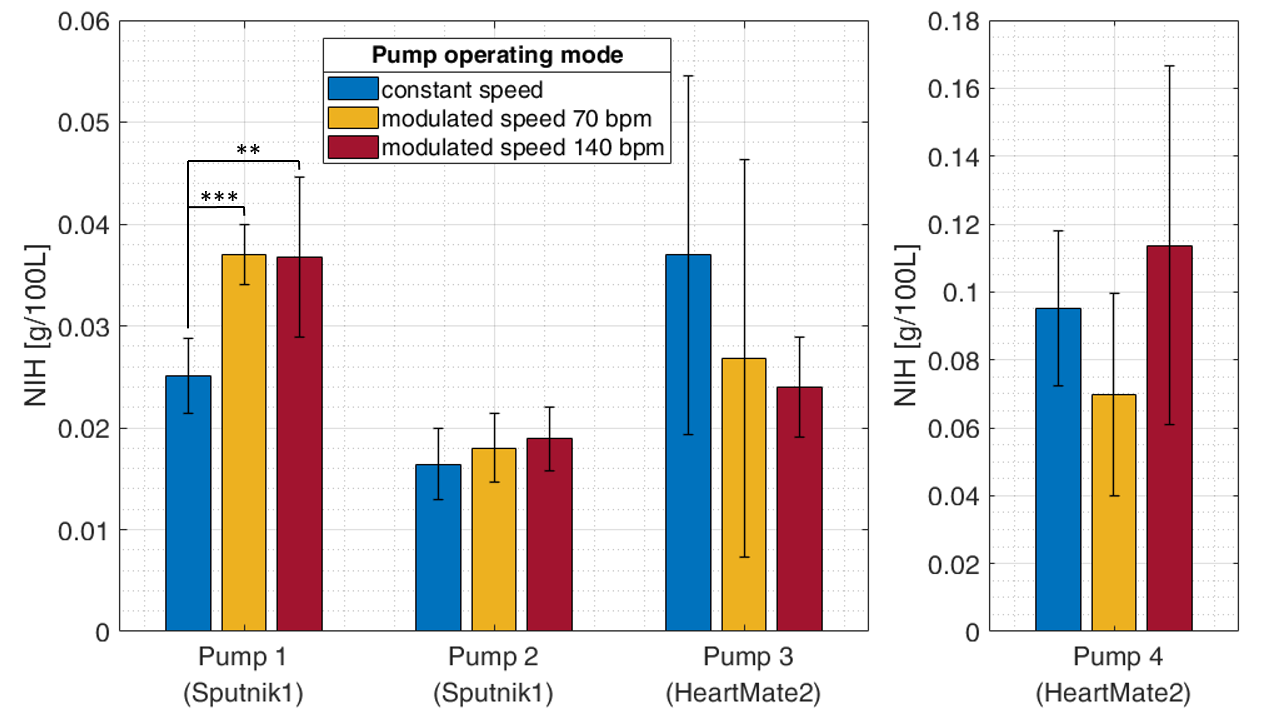


**Figure 1.** The normalized index of hemolysis (NIH) for all operating modes and all pumps. Significance was tested for CS mode compared to both MS modes. *p < 0.05; **p < 0.01; ***p < 0.001.

1. Parameters measured with cell counter


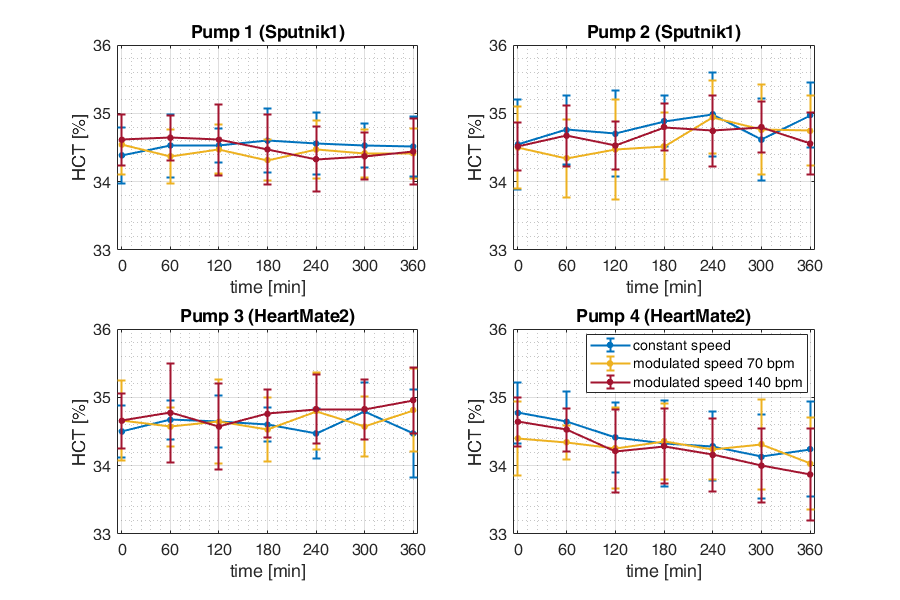


**Figure 2.** Hematocrit (HCT) over time for all operating modes and all pumps. Significance was tested for CS mode compared to both MS modes. *p < 0.05; **p < 0.01; ***p < 0.001.

**
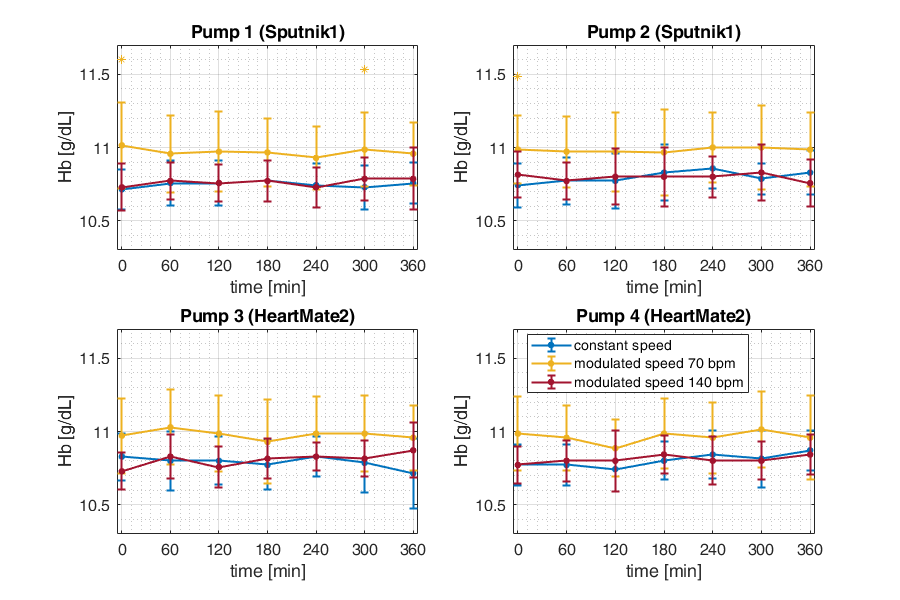
Figure 3.** Total hemoglobin concentration (Hb) over time for all operating modes and all pumps. Significance was tested for CS mode compared to both MS modes. *p < 0.05; **p < 0.01; ***p < 0.001.


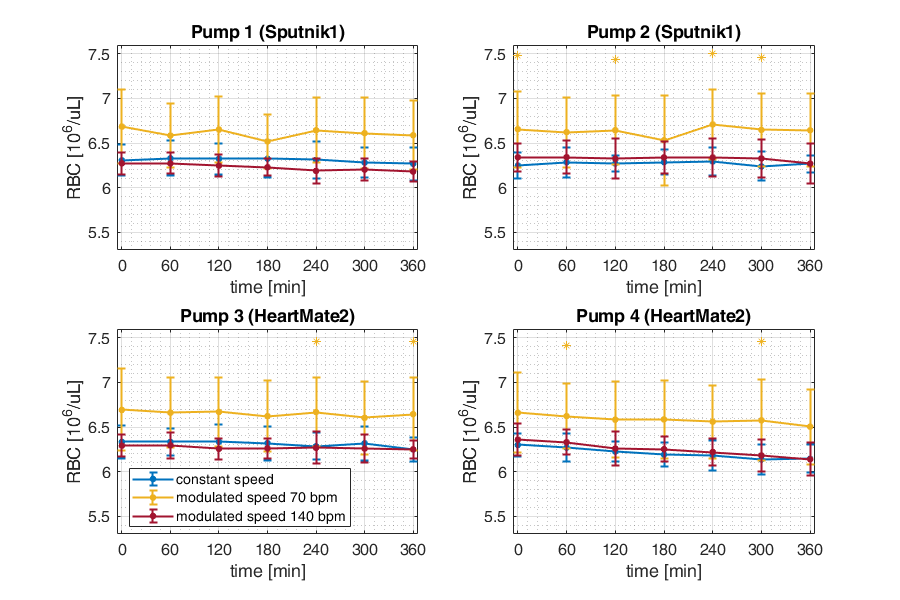


**Figure 4.** Red blood cell count (RBC) over time for all operating modes and all pumps. Significance was tested for CS mode compared to both MS modes. *p < 0.05; **p < 0.01; ***p < 0.001.


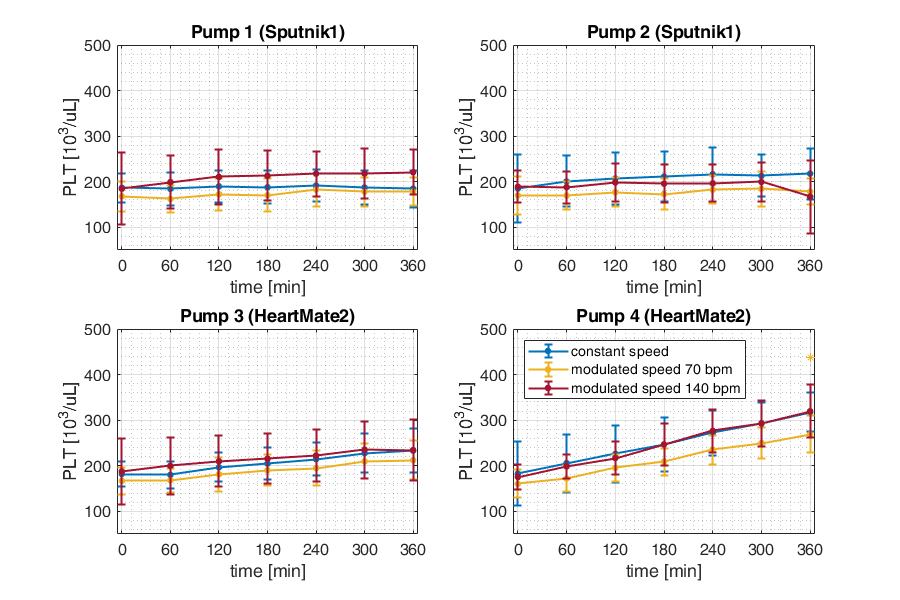


**Figure 5.** Platelet count (PLT) over time for all operating modes and all pumps. Significance was tested for CS mode compared to both MS modes. *p < 0.05; **p < 0.01; ***p < 0.001.


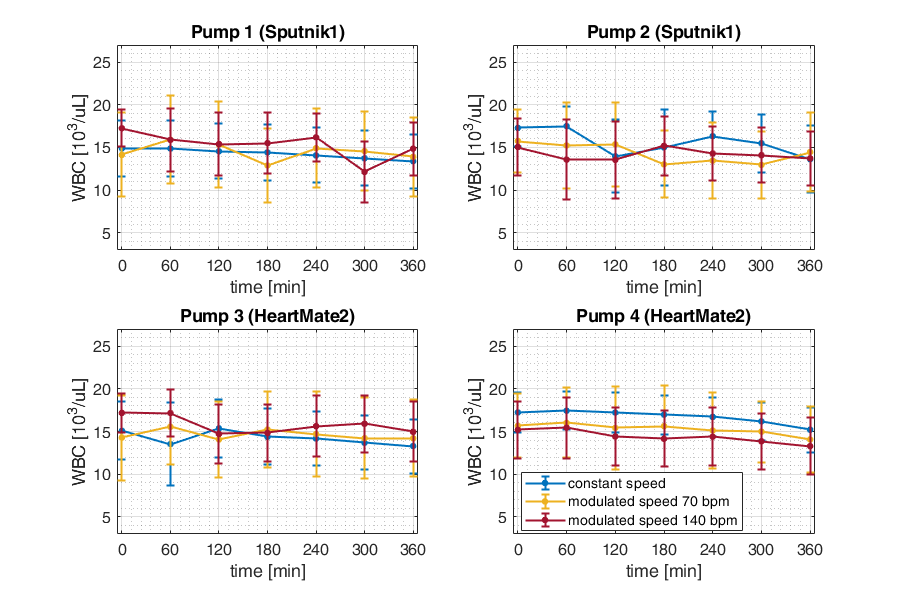


**Figure 6.** White blood cell count (WBC) over time for all operating modes and all pumps. Significance was tested for CS mode compared to both MS modes. *p < 0.05; **p < 0.01; ***p < 0.001.

1. Parameters measured with blood gas analysis device:


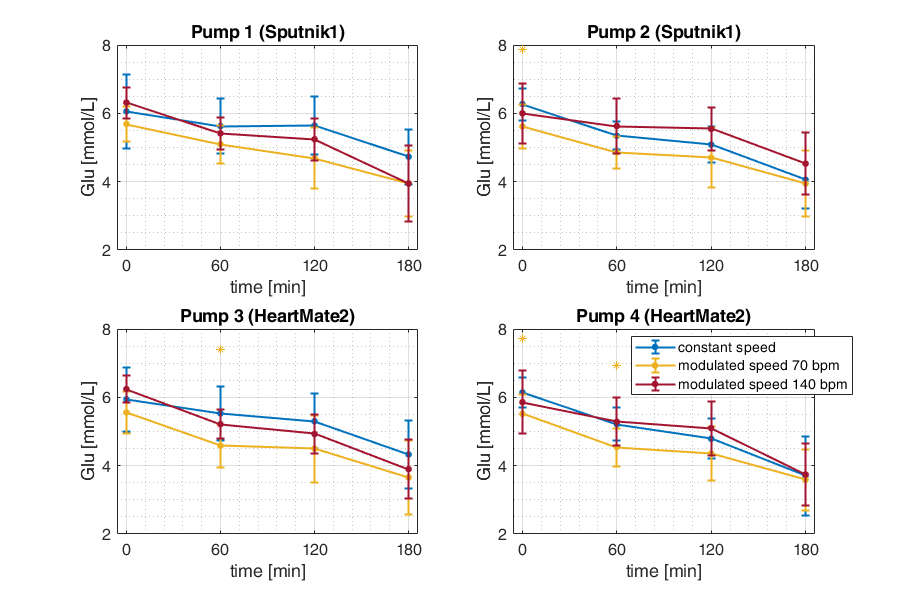


**Figure 7.** Blood glucose (Glu) over time for all operating modes and all pumps. Significance was tested for CS mode compared to both MS modes. *p < 0.05; **p < 0.01; ***p < 0.001.


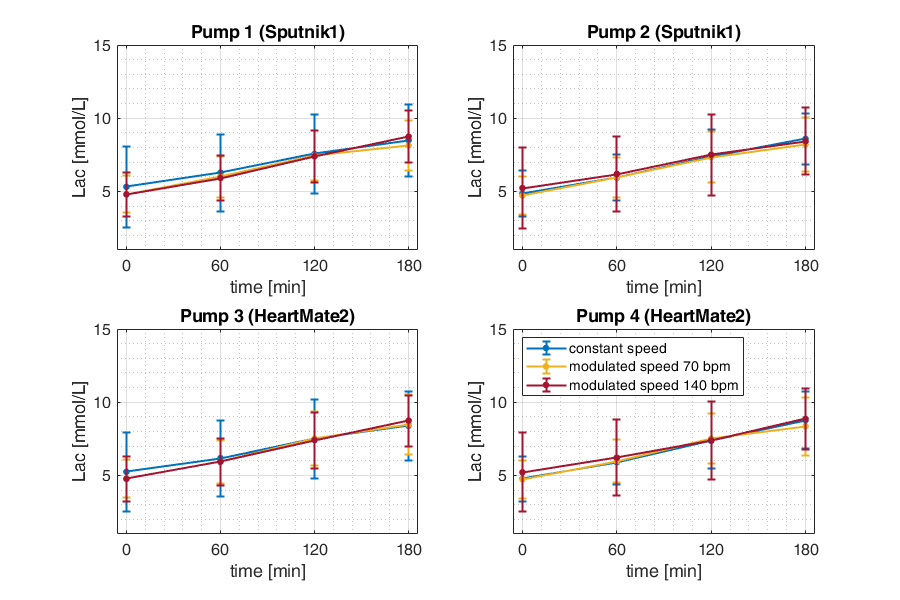


**Figure 8.** Lactate concentration (Lac) over time for all operating modes and all pumps. Significance was tested for CS mode compared to both MS modes. *p < 0.05; **p < 0.01; ***p < 0.001.


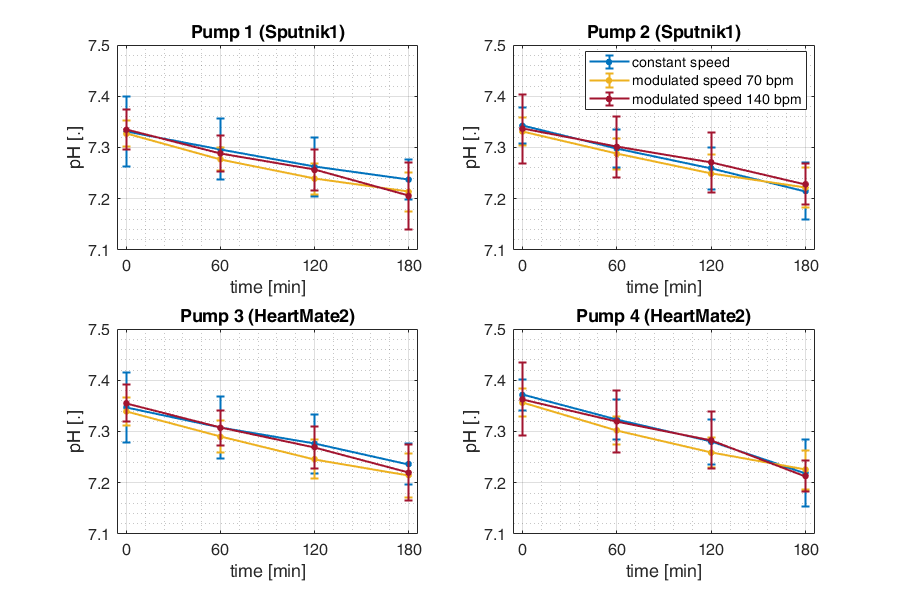


**Figure 9.** pH value over time for all operating modes and all pumps. Significance was tested for CS mode compared to both MS modes. *p < 0.05; **p < 0.01; ***p < 0.001.


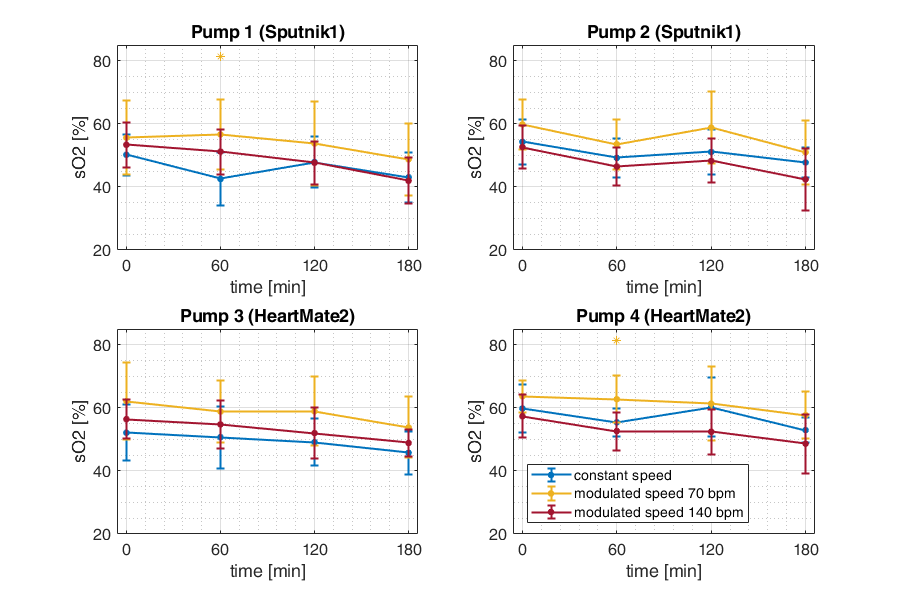


**Figure 10.** Oxygen saturation (sO2) over time for all operating modes and all pumps. Significance was tested for CS mode compared to both MS modes. *p < 0.05; **p < 0.01; ***p < 0.001.


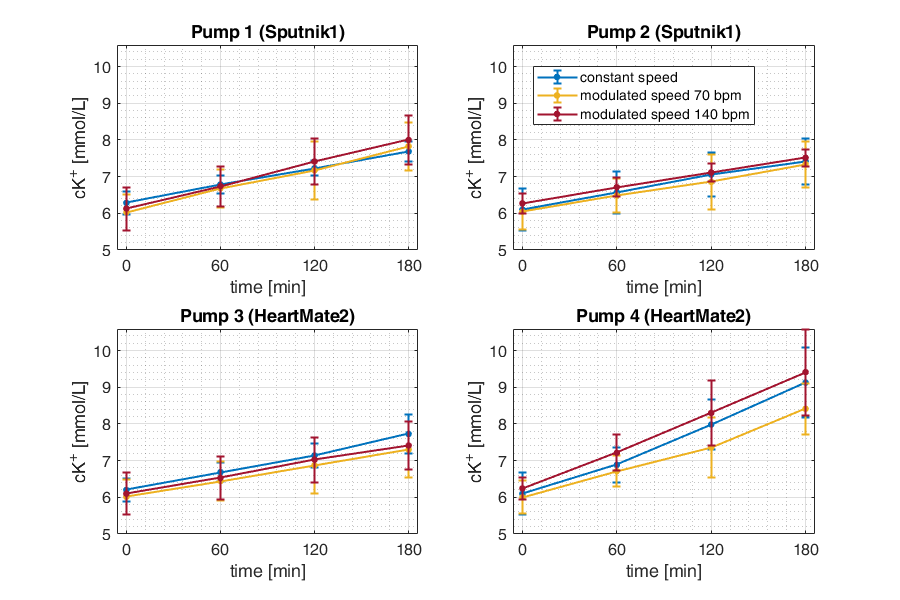


**Figure 11.** Potassium concentration (cK^+^) over time for all operating modes and all pumps. Significance was tested for CS mode compared to both MS modes. *p < 0.05; **p < 0.01; ***p < 0.001.
